# Alexithymia and its subgroup characteristics in Chinese empty-nesters with multiple chronic diseases: an exploration based on latent class analysis

**DOI:** 10.3389/fpsyt.2026.1779610

**Published:** 2026-05-12

**Authors:** Bin Shang, Jianhua Li, Ming Dai, Zhengdong Ge, Caifeng Luo, Jing Wu, Xiao Shao, Yan Xu

**Affiliations:** 1 Department of Nursing, The First People’s Hospital of Lianyungang, Lianyungang, China; 2 School of Medicine, Jiangsu University, Zhenjiang, China; 3 University Hospital, Nanjing University of Aeronautics and Astronautics, Changzhou, China; 4 Endoscopy Center, Suqian First People’s Hospital, Suqian, China

**Keywords:** alexithymia, elderly, empty-nest elders, latent class analysis, multiple chronic conditions

## Abstract

**Background:**

Alexithymia is common among older adults, particularly those with multiple chronic conditions; however, its heterogeneity in empty-nest populations remains insufficiently explored. This study aimed to identify latent subgroups of alexithymia and examine their characteristics among elderly empty-nesters with multiple chronic diseases.

**Methods:**

A total of 561 empty-nest older adults with at least two chronic conditions were recruited from six cities in Jiangsu Province, China (November 2022–March 2024). Alexithymia was assessed using the Toronto Alexithymia Scale (TAS-20), and latent class analysis was conducted to identify subgroups.

**Results:**

The total alexithymia score of the empty-nest elderly with multiple chronic diseases was (57.31 ± 9.80) points. Alexithymia can be divided into four potential subgroups: the Emotional Repression Group (39.2%), the Extraverted Thinking Group (11.6%), the Emotional Ambiguity Group (10.2%), and the Good Emotional Adaptation Group (39.0%). Multiple chronic diseases among empty-nesters in different alexithymia subgroups were statistically significant in terms of age group, average monthly household income, whether an acute event had occurred within the past year, and perception of their own health.

**Conclusion:**

Alexithymia in elderly empty-nesters with multiple chronic diseases shows distinct patterns. Medical staff should focus on the Emotional Ambiguity and Emotional Repression groups and provide targeted interventions for each type.

## 1 Introduction

With the rapid development of China’s social economy, its population structure has experienced significant changes. According to the seventh national census, 18.7% of the population, approximately 264 million people, are aged 60 and above (1). The aging population is becoming a more pressing issue, presenting new challenges to the physical and mental health of the elderly. Among these challenges, the issue of empty-nesters stands out. Empty-nesters are elderly individuals who live alone or with their spouses, without the presence of their children. As urbanization progresses, the traditional family structure has shifted, with children moving away for work or study. The seventh national census predicts that by 2030, the number of empty-nesters in China will exceed 200 million, making this the dominant family model for the elderly (2, 3). This trend indicates that the mental health problems of empty-nesters will intensify, requiring urgent attention and intervention from society.

Empty-nesters often lack emotional support from their children, making them more likely to experience anxiety and depression, which hinder their active aging (4). Studies show that emotional loneliness and social isolation are common in this group (5). The absence of family support makes them feel more helpless when facing challenges. Chronic diseases increase the risk of psychological problems, threatening both physical and mental health (4). Additionally, living in an empty-nest state raises the risk of cognitive decline in those with chronic diseases (6). Emotional isolation and multiple health issues worsen their distress, especially when their physical health declines. Research shows that older adults with multiple chronic diseases experience more psychological distress than those with a single chronic illness, with a higher likelihood of depression, anxiety, and loneliness (7). Identifying and addressing these issues is key to promoting active aging. However, few studies focus on empty-nesters with multiple chronic diseases, and the impact of alexithymia is often overlooked.

As an affective cognitive disorder, alexithymia is usually manifested as an individual’s difficulty in recognising and expressing their emotions and feelings (8). Alexithymia can be divided into primary and secondary types. Primary alexithymia is usually a stable personality trait, in which individuals have difficulty recognising and expressing emotions since childhood, and this disorder remains relatively constant in adulthood. However, with age, especially under the influence of factors such as psychological stress, chronic illness or emotional deprivation, some older people may develop secondary alexithymia (9, 10). Studies have shown that older adults with multiple chronic diseases are more likely to develop alexithymia than those with a single chronic disease. This may be closely related to the stress of disease management, changes in physical health, and emotional isolation (11). Alexithymia not only affects older adults’ perception of their emotions, but may also interfere with their perception of self-aging, further increasing the risk of psychological symptoms such as anxiety and depression (12). Therefore, effectively identifying the influencing factors of alexithymia in the group of empty-nest elderly people with multiple chronic diseases is of great significance for improving their emotional health and psychological intervention.

However, previous studies (13) mostly based on the total score to determine the level of alexithymia of individuals, ignoring the individual differences of the population, which may lead to distorted results. Latent class analysis (LCA) provides a new way of thinking to solve this problem (14). Latent class analysis explains the relationship between external categorical variables through latent class variables and achieves external independence between manifest variables. The latent class of individuals can be determined, and objective statistical indicators can be used to measure the accuracy and validity of the classification, thereby ensuring that intergroup differences and intragroup homogeneity are maximised (15). Latent class analysis is widely used in the study of psychological problems in the elderly. For example, Se (16) and others used multiple group LCA to identify latent subgroups of mental health and cognitive symptoms in the elderly. Liu (17) and others revealed the characteristics of latent subgroups of social isolation and depression in the elderly with different functional states. These studies have focused on multiple dimensions of psychological problems in older adults. However, there has been limited attention paid to empty-nest older adults with multiple chronic conditions, especially their alexithymia potential subgroup. The effective identification of their potential subgroup is of great significance for improving the emotional state and promoting the mental health of older adults.

Therefore, this study aims to use latent class analysis to classify potential subgroups of alexithymia in empty-nested elderly people with multiple chronic diseases, explore the differences and influencing factors between different potential subgroups, put forward targeted suggestions based on the characteristics of different potential subgroups of alexithymia, and provide a reference for medical personnel to designate targeted interventions in the later stage.

## 2 Methods

### 2.1 Study design and participants

This multi-centre cross-sectional study was conducted between November 2022 and March 2024, mainly using a convenience sampling method. The sample was derived from older adults with multiple chronic diseases in communities and villages in six prefecture-level cities in Jiangsu Province, China (Nanjing, Suzhou, Zhenjiang, Changzhou, Lianyungang, and Suqian). The sample selected this time involves the southern, central, and northern Jiangsu provinces, so it is quite representative. Inclusion criteria: (1) age ≥ 60 years; (2) suffering from two or more chronic diseases, with the scope of comorbidity using the types of chronic diseases provided in the Charlson Comorbidity Index; (3) elderly people living alone or with their spouse; (4) informed consent and voluntary participation in this study. Exclusion criteria: (1) elderly people who are unwilling to participate in the study; (2) elderly people with cognitive dysfunction or intellectual problems that may affect the informed validity of this study; (3) non-community residents or residents whose place of residence cannot be determined; (4) those who are participating in other research projects. Nylund-Gibson (18) suggests that the minimum sample size for latent class analysis is 300 cases. This study actually included 561 cases.

Data collection used a combination of online and offline methods. For the online collection part, a questionnaire was posted on the online questionnaire collection platform Wenjuanxing (www.wjx.cn). For illiterate or elderly people who were unable to complete the questionnaire successfully, the researcher asked questions orally and filled in the online questionnaire after checking with the research subject that the information was correct. Previous research experience tells us that using multiple sample collection methods can better avoid missing key samples. Considering that there are also elderly people who do not have a smartphone but still want to complete the questionnaire on their own, we have also prepared some paper questionnaires that are the same as the online questionnaires, and the filling requirements are the same as those for the online questionnaires. This research plan is in line with the Helsinki Declaration and has been reviewed and approved by the Medical Ethics Committee of Jiangsu University, with approval number No. 20221019-7.

### 2.2 Measurements

#### 2.2.1 Demographic information and disease characteristics

The demographic and disease-related information collected in this study includes gender, age, marital status, residence, residence status, education level, average monthly household income, financial pressure of illness, number of chronic diseases, and polypharmacy(≥5), medical insurance payment method, frequency of visits by children, whether an acute event (e.g., hospitalization, acute trauma, etc.) occurred within the past year, and perception of one’s own health status.

#### 2.2.2 The Toronto alexithymia scale

The Chinese version of the Toronto Alexithymia Scale (TAS-20) was used to assess participants’ alexithymia levels. Originally developed by Taylor et al. (8), the scale was later cross-culturally adapted by Yijin Yao et al. (19) to create the Chinese version. The Cronbach’s alpha coefficient for the total scale is 0.830. The scale consists of 20 items covering three dimensions: difficulty identifying feelings (DIF, items1, 3, 6, 7, 9, 13, 14); difficulty describing feelings (DDF, items 2, 4, 11, 12, 17); and externally oriented thoughts(EOTS, items 5, 8, 10, 15, 16, 18, 19, 20). For detailed items information, see **Supplementary Table 1**. It uses a 5-point Likert scale ranging from 1 (strongly disagree) to 5 (strongly agree). The total score ranges from 20 to 100, with higher scores indicating higher levels of alexithymia. A score of ≥61 is used to identify the presence of alexithymia. In this study, the Cronbach’s alpha coefficient for the total scale was 0.884.

### 2.3 Statistical analysis

A latent class analysis was performed using Mplus 8.0. First, the data measured by the alexithymia scale were converted to binary variables (0 and 1). Raw scores of 4 or higher (including “strongly agree” and “agree”) were classified as indicating alexithymia and assigned a value of 1, whereas raw scores of 3 or lower (including “strongly disagree, “ “disagree, “ and “neutral”) were classified as not indicating alexithymia and assigned a value of 0. This dichotomization was applied to facilitate latent class modelling using binary indicators and to enhance the interpretability of subgroup characteristics. In this approach, higher response categories were treated as more definitive endorsements of alexithymia-related traits, allowing clearer differentiation between individuals with more pronounced versus less pronounced emotional difficulties. Although responses in the mid-range (e.g., “neutral”) may reflect more ambiguous emotional states, classifying them into the lower category was intended to improve classification clarity. However, this transformation may lead to a loss of information and reduced variability, and the results should be interpreted with this limitation in mind. Starting with a single-category initial model, the number of categories is gradually increased to determine the best model. Model fit tests include the Akaike Information Criterion (AIC), the Bayesian Information Criterion (BIC), and the Adjusted Bayesian Information Criterion (aBIC). The smaller the value, the better the model fit. The entropy value is used to assess the accuracy of classification, with a value of 0 to 1. The closer it is to 1, the higher the classification accuracy. A value of approximately 0.8 indicates a classification accuracy of over 90%. Likelihood ratio test indicators include Lo‐Mendell‐Rubin (LMR) and Bootstrap‐based likelihood ratio (BLRT), which are used to compare the differences in the goodness of fit of potential category models. P<0.05 indicates that the model with K categories is superior to the model with K-1 categories (15). Data analysis and processing were performed using SPSS 26.0. The chi-square test and analysis of variance were used for comparisons between categories, and logistic regression was used for multiple factor analysis. The level of significance was α=0.05.

## 3 Results

### 3.1 Alexithymia scores of empty-nesters with multiple chronic diseases

The results showed that the total alexithymia score of the elderly with chronic diseases and comorbidities was (57.31 ± 9.80) points, and the average score of the entries was (2.87 ± 0.49) points; the affective identification dimension was (20.02 ± 5.12) points, and the average score of the entries was (2.86 ± 0.73) points; the affective description dimension was (14.47 ± 3.04) points, item mean score (2.89 ± 0.61) points; extraverted thinking dimension (22.82 ± 3.05) points, item mean score (2.85 ± 0.38) points;. The prevalence of alexithymia in elderly people with chronic diseases was 41.4%. See **Table 1** for details.

**Table 1:** Alexithymia scores of empty-nesters with multiple chronic diseases. (n=561, X ± S, points).

Variables	Items	Score range	Score	Average score of items
Total score	20	20~100	57.31 ± 9.80	2.87 ± 0.49
Difficulty Identifying Feelings	7	7~35	20.02 ± 5.12	2.86 ± 0.73
Difficulty Describing Feelings	5	5~25	14.47 ± 3.04	2.89 ± 0.61
Externally Oriented Thoughts	8	5-40	22.82 ± 3.05	2.85 ± 0.38

### 3.2 Empty-nesters with multiple chronic conditions alexithymia potential subgroup

This study fitted a total of five latent class models, as shown in **Table 2**. With the increase in the number of classes, the Akaike information criterion (AIC), Bayesian information criterion (BIC), and adjusted Bayesian information criterion (aBIC) values decreased, indicating improved model fit. However, for the five-class model, the Lo–Mendell–Rubin (LMR) likelihood ratio test was not statistically significant (P > 0.05), suggesting that the five-class solution did not provide a significantly better fit than the four-class model. In addition, the entropy value decreased in the five-class model, indicating reduced classification accuracy.

**Table 2:** Empty-nesters with multiple chronic diseases alexithymia in the category analysis of each model fitting index.

Models	AIC	BIC	aBIC	Entropy	LMR (*P*)	BLRT (*P*)	Category probability
1	12247.503	12334.097	12270.608	–	–	–	1
2	10530.703	10708.221	10578.067	0.893	<0.001	<0.001	0.373/0.627
3	9999.091	10267.533	10070.715	0.906	<0.001	<0.001	0.102/0.392/0.506
4	9891.469	10250.836	9987.352	0.882	0.001	<0.001	0.392/0.116/0.102/0.390
5	9865.704	10315.995	9985.847	0.838	0.183	<0.001	0.102/0.168/0.241/0.116/0.374

Akaike information criterion (AIC), Bayesian information criterion (BIC), Adjusted Bayesian information criterion (aBIC), Entropy, Lo-Mendell-Rubin likelihood (LMR), Bootstrapped likelihood ratio test (BLRT).

**Figure 1:**
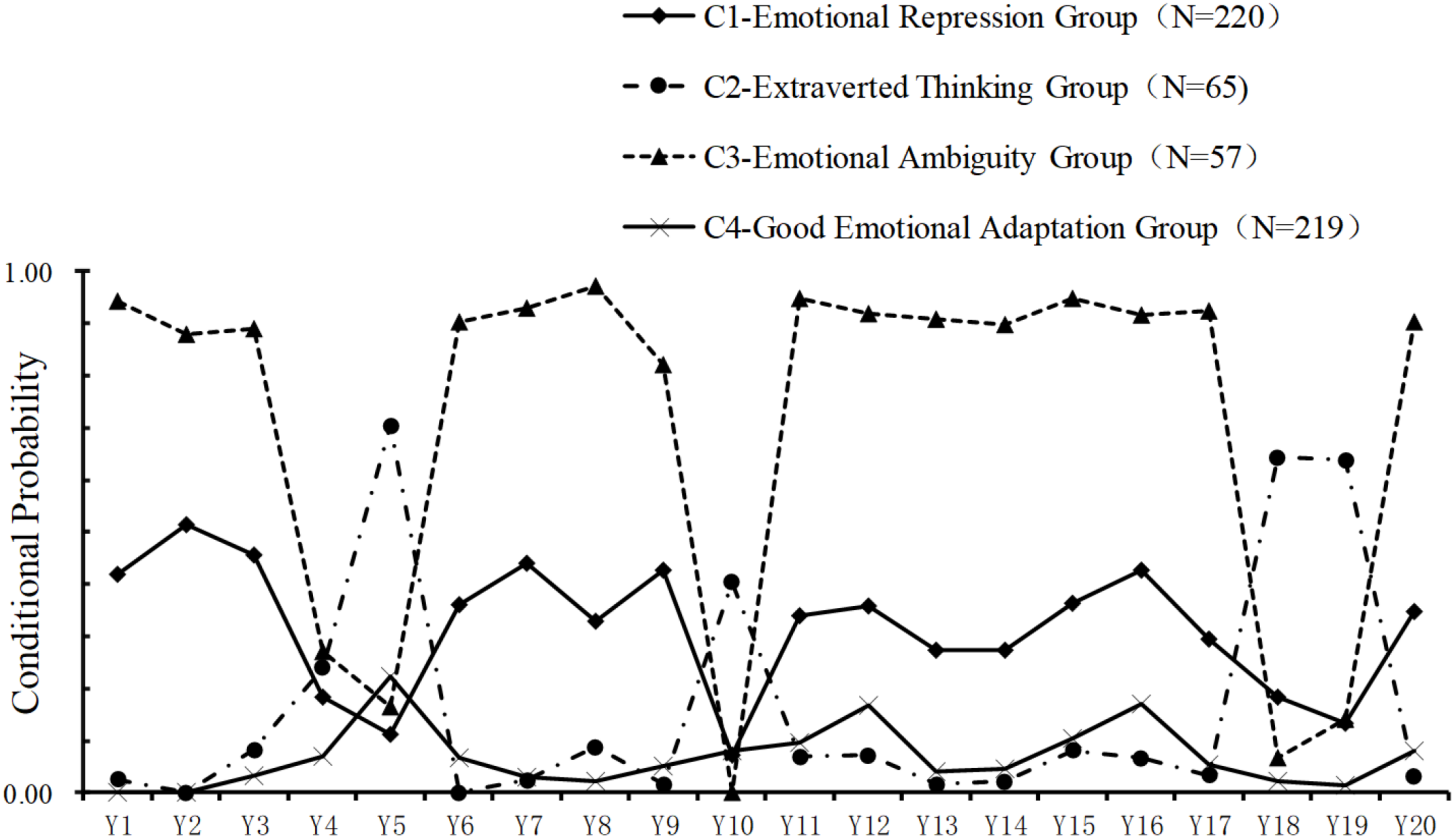
Empty-nesters with multiple chronic conditions alexithymia probability distribution of conditions for the four latent categories; for detailed items information, see **Supplementary Table 1**.

Considering these statistical indicators, together with classification performance, model parsimony, and interpretability, the four-class model was selected as the optimal solution. The average posterior probabilities for class membership in the four-class model were 95.3%, 90.1%, 97.0%, and 92.3%, respectively, indicating good classification quality. The conditional probabilities of the four classes across the 20 items are presented in **Figure 1**. Category 1 (C1) contains a total of 220 cases. This category scores similarly on Difficulty Identifying Feelings and Difficulty Describing Feelings and both are at a medium level, suggesting that these individuals may suppress their emotions and have difficulty regulating them through extroverted thinking. Therefore, it is named the ‘Emotional Repression Group’. The second category (C2) contains a total of 65 cases. This category has low scores in Difficulty Identifying Feelings and Difficulty Describing, and significantly higher scores in extroverted thinking, indicating that individuals in this category have strong extroverted thinking characteristics and tend to regulate emotions through extroverted thinking. Therefore, it is named the ‘Extraverted Thinking Group’. The third group (C3) contains 57 cases. This group scored higher on all three dimensions, so it was named the ‘Emotional Ambiguity Group’. The fourth group (C4) contains 219 cases. The scores in this group are relatively balanced and lower across all dimensions, indicating that individuals can better adapt to the recognition and expression of emotions and the regulation of extroverted thinking. They are emotionally healthy and well-adjusted, hence the name ‘Good Emotional Adaptation Group’. The alexithymia scores for the four categories were (62.20 ± 5.07) points, (51.68 ± 5.97) points, (73.05 ± 4.16) points and (49.97 ± 7.33) points respectively.

### 3.3 Comparison of alexithymia in elderly empty nesters with multiple chronic diseases of different characteristics

The results of the univariate analysis showed that there was no statistically significant difference (P>0.05) in the comparison of the alexithymia categories of empty-nesters with chronic diseases co-morbidity of different genders and different types of medical insurance. This approach was used as an initial screening step and was interpreted in conjunction with theoretical considerations and previous literature. There was a statistically significant difference (P<0.05) in the category comparison of psychological distress in the elderly with chronic diseases who age group, marital status, residence, residence status, education level, average monthly household income, financial pressure of illness, number of chronic diseases, and polypharmacy(≥5), frequency of visits by children, whether an acute event (e.g., hospitalization, acute trauma, etc.) occurred within the past year, and perception of one’s own health status. See **Table 3**.

**Table 3:** Comparison of alexithymia categories among empty-nesters with multiple chronic diseases of different demographic characteristics (n=561).

Variables	Emotional repression group (*n* = 220)	Extraverted thinking group (*n* = 65)	Emotional ambiguity group (*n* = 57)	Good emotional adaptation group (*n* = 219)	*χ^2^-value*	*P-value*
Gender					1.833	0.608
Male	135 (61.36)	35 (53.85)	34 (59.65)	123 (56.16)		
Female	85 (38.64)	30 (46.15)	23 (40.35)	96 (43.84)		
Age group					76.534	<0.001
60–69 years old	76 (34.55)	48 (73.85)	16 (28.07)	135 (61.64)		
70–79 years old	107 (48.64)	11 (16.92)	24 (42.10)	76 (34.70)		
≥80 years old	37 (16.81)	6 (9.23)	17 (29.83)	8 (3.65)		
Residence					17.652	<0.001
Rural	140 (63.64)	35 (53.85)	45 (78.95)	112 (51.14)		
Urban	80 (36.36)	30 (46.15)	12 (21.05)	107 (48.86)		
Marital Status					31.873	<0.001
Married	188 (85.46)	61 (93.84)	41 (71.93)	204 (93.15)		
Widowed	26 (11.82)	2 (3.08)	14 (24.56)	7 (3.20)		
Other	6 (2.72)	2 (3.08)	2 (3.51)	8 (3.65)		
Education level					32.643	<0.001
Primary school and below	101 (45.91)	25 (38.46)	40 (70.18)	73 (33.33)		
Middle school	79 (35.91)	25 (38.46)	11 (19.30)	93 (42.47)		
High school	25 (11.36)	12 (18.46)	5 (8.77)	44 (20.09)		
College and above	15 (6.82)	3 (4.62)	1 (1.75)	9 (4.11)		
Average monthly household income					51.926	<0.001
<3000CNY	96 (43.64)	24 (36.92)	43 (75.44)	68 (31.05)		
3000–5000 CNY	77 (35.00)	26 (40.00)	8 (14.04)	96 (43.84)		
5000–8000 CNY	36 (16.36)	7 (10.77)	4 (7.02)	49 (22.37)		
8000 or more CNY	11 (5.00)	8 (12.31)	2 (3.50)	6 (2.74)		
Financial pressure due to illness					31.564	<0.001
Relatively high	35 (15.91)	10 (15.39)	21 (36.84)	24 (10.96)		
Average	148 (67.27)	50 (76.92)	26 (45.61)	171 (78.08)		
Relatively low	37 (16.82)	5 (7.69)	10 (17.54)	24 (10.96)		
Number of chronic diseases					27.902	<0.001
2 types	89 (40.45)	30 (46.15)	20 (35.09)	114 (52.05)		
3 types	99 (45.00)	31 (47.69)	20 (35.09)	89 (40.64)		
4 types and above	32 (14.55)	4 (6.15)	17 (29.82)	16 (7.31)		
polypharmacy					21.208	<0.001
Yes	41 (18.64)	8 (12.31)	23 (40.35)	33 (15.07)		
No	179 (81.36)	57 (87.69)	34 (59.65)	186 (84.93)		
Medical insurance payment method					10.420	0.318
Out-of-pocket expenses	8 (3.64)	1 (1.53)	2 (3.50)	6 (2.74)		
New Rural Cooperative Medical System	134 (60.91)	41 (63.08)	40 (70.18)	117 (53.42)		
Urban and rural residents medical insurance	56 (25.45)	14 (21.54)	9 (15.79)	59 (26.94)		
Employee medical insurance	22 (10.00)	9 (13.85)	6 (10.53)	37 (16.90)		
Residence Status					8.904	0.031
Living alone	62 (28.18)	15 (23.08)	21 (36.84)	43 (19.63)		
Living with spouse	158 (71.82)	50 (76.92)	36 (63.16)	176 (80.37)		
Frequency of children’s visits					31.304	<0.001
4 or more times per month	66 (30.00)	20 (30.77)	15 (26.32)	90 (41.10)		
1 or more times per month	42 (19.09)	13 (20.00)	7 (12.28)	42 (19.18)		
Less than once a month	77 (35.00)	18 (27.69)	12 (21.05)	59 (26.94)		
Less than once a year	35 (15.91)	14 (21.54)	23 (40.35)	28 (12.78)		
Acute events within 1 year					44.912	<0.001
Yes	123 (55.91)	20 (30.77)	35 (61.40)	63 (28.77)		
No	97 (44.09)	45 (69.23)	22 (38.60)	156 (71.23)		
Perception of health					89.800	<0.001
Poor	80 (36.36)	6 (9.23)	33 (57.90)	23 (10.50)		
Fair	124 (56.37)	52 (80.00)	22 (38.60)	153 (69.86)		
Good	16 (7.27)	7 (10.77)	2 (3.50)	43 (19.64)		

### 3.4 Empty-nesters with multiple chronic diseases alexithymia Multiclass logistic regression analysis of influencing factors

The potential category of alexithymia in empty-nest elderly with multiple chronic diseases is used as the dependent variable, and the ‘Good Emotional Adaptation Group’ is used as the reference group. The independent variable assignment method is shown in **Table 4**. The indicators withP <0.05 in the univariate analysis were included as independent variables in the multivariate logistic regression. Multicollinearity diagnostics indicated that all variance inflation factors (VIFs) were below 5, suggesting no evidence of significant multicollinearity among the independent variables. In addition, the likelihood ratio test showed that the model was statistically significant (χ^2^ = 224.802, df = 69, P < 0.001), indicating that the independent variables collectively contributed to the model. The results showed that compared with the ‘Good Emotional Adaptation Group’, those aged 70 to 79 (OR = 1.809, P = 0.011), ≥80 years old (OR = 4.472, P = 0.002), those who had an acute event within the past year (OR = 2.018, P = 0.003), empty nesters with multiple chronic diseases who perceive their health as fair (OR = 2.806, P = 0.043) or poor (OR = 6.168, P<0.001) are more likely to be in the ‘Emotional Repression Group’;

**Table 4:** Assignment of independent variables.

Variables	Assignment
Age group	60–69 years old (X_1_ = 0, X_2_ = 0); 70–79 years old (X_1_ = 1, X_2_ = 0); ≥80 years old (X_1_ = 0, X_2_ = 1);
Residence	Urban (X_1_ = 0); Rural (X_1_ = 1);
Marital Status	Other (X_1_ = 0, X_2_ = 0); Married (X_1_ = 1, X_2_ = 0); Widowed (X_1_ = 0, X_2_ = 1);
Education level	College and above (X_1_ = 0, X_2_ = 0, X_3_ = 0); Primary school and below (X_1_ = 1, X_2_ = 0, X_3_ = 0); Middle school (X_1_ = 0, X_2_ = 1, X_3_ = 0); High school (X_1_ = 0, X_2_ = 0, X_3_ = 1);
Average monthly household income	<3000 CNY (X_1_ = 0, X_2_ = 0, X_3_ = 0); 3000–5000 CNY(X_1_ = 1, X_2_ = 0, X_3_ = 0); 5000–8000 CNY(X_1_ = 0, X_2_ = 1, X_3_ = 0); 8000 or more CNY (X_1_ = 0, X_2_ = 0, X_3_ = 1);
Financial pressure due to illness	Relatively low (X_1_ = 0, X_2_ = 0); Average (X_1_ = 1, X_2_ = 0); Relatively high (X_1_ = 0, X_2_ = 1);
Number of chronic diseases	2 types (X_1_ = 0, X_2_ = 0); 3 types (X_1_ = 1, X_2_ = 0); 4 types and above (X_1_ = 0, X_2_ = 1);
Polypharmacy	No (X_1_ = 0); Yes(X_1_ = 1),;
Residence Status	Living alone (X_1_ = 0); Living with spouse (X_1_ = 1);
Frequency of children’s visits	4 or more times per month (X_1_ = 0, X_2_ = 0, X_3_ = 0); 1 or more times per month (X_1_ = 1, X_2_ = 0, X_3_ = 0); Less than once a month (X_1_ = 0, X_2_ = 1, X_3_ = 0); Less than once a year (X_1_ = 0, X_2_ = 0, X_3_ = 1);
Acute events within 1 year	No (X_1_ = 0); Yes (X_1_ = 1);
Perception of health	Good (X_1_ = 0, X_2_ = 0); Fair (X_1_ = 1, X_2_ = 0); Poor (X_1_ = 0, X_2_ = 1);

Compared with the ‘Good Emotional Adaptation Group’, empty-nesters with multiple chronic diseases and a monthly household income of ≥8, 000 CNY (OR = 5.915, P = 0.020) were more likely to be classified in the ‘Extraverted Thinking Group’, while individuals aged 70–79 (OR = 0.372, P = 0.012) were more likely to be classified in the ‘Good Emotional Adaptation Group’.

Compared to the ‘Good Emotional Adaptation Group, ‘older adults aged ≥80 years (OR = 7.869, P = 0.001) and those who perceive their health status to be poor are more likely to be classified as belonging to the ‘Emotional Ambiguity Group.’ Individuals with a family average monthly income of 3, 000-5, 000 CNY (OR = 0.329, P = 0.024) and 5, 000-8, 000 CNY (OR = 0.246, P = 0.049) were more likely to belong to the ‘Good Emotional Adaptation Group’; see **Table 5** for details.

**Table 5:** Logistic regression of alexithymia categories influencing factors in empty-nest elderly people with multiple chronic diseases (n=561).

Variables	Emotional repression group				Extraverted thinking group				Emotional ambiguity group			
	*β*	OR	95%*CI*	*P*-value	*β*	OR	95%*CI*	*P*-value	*β*	OR	95%*CI*	*P*-value
Age group												
60–69 years old												
70–79 years old	0.593	1.809	1.145-2.856	0.011	-0.989	0.372	0.172-0.805	0.012	0.468	1.597	0.716-3.56	0.253
≥80 years old	1.498	4.472	1.742-11.478	0.002	0.580	1.787	0.472-6.769	0.393	2.063	7.869	2.208-28.043	0.001
Average monthly household income												
<3000CNY												
3000–5000 CNY	-0.014	0.986	0.579-1.681	0.959	-0.123	0.884	0.429-1.821	0.738	-1.111	0.329	0.126-0.862	0.024
5000–8000 CNY	-0.090	0.914	0.442-1.892	0.809	-0.666	0.514	0.172-1.536	0.233	-1.404	0.246	0.06-0.997	0.049
8000 or more CNY	0.859	2.361	0.649-8.584	0.192	1.778	5.915	1.32-26.513	0.020	0.147	1.158	0.137-9.784	0.893
Acute events within 1 year												
No												
Yes	0.702	2.018	1.263-3.223	0.003	0.183	1.201	0.608-2.373	0.598	0.074	1.077	0.486-2.389	0.855
Perception of health												
Good												
Fair	0.735	2.086	1.022-4.258	0.043	0.985	2.678	0.97-7.395	0.057	1.452	4.271	0.836-21.816	0.081
Poor	1.819	6.168	2.536-15.002	0.000	0.722	2.059	0.494-8.588	0.322	3.100	22.197	3.739-131.761	0.001

## 4 Discussion

To our knowledge, this is the first study to focus on the characteristics of different potential subgroups of Chinese empty-nesters with multiple chronic diseases and alexithymia. We used latent class analysis to group according to alexithymia and identified four different subgroups, which were heuristically named according to their item characteristics: the Emotional Repression Group (C1), the Extraverted Thinking Group (C2), the Emotional Ambiguity Group (C3) and the Good Emotional Adaptation Group (C4), with significant heterogeneity between the groups.

Participants in the Emotional Repression Group (C1) accounted for 39.2%, the highest proportion among the four subgroups, with an average alexithymia score (62.20 ± 5.07). Comparisons within the group found that the average alexithymia score of participants in this group was at a medium to high level on all items. The highest score was for item 2, ‘I have difficulty finding the right words to describe how I feel’. This indicates that this group of older adults has problems expressing their feelings and is prone to emotional suppression. Chronic diseases and empty-nest status have been reported to be associated with increased psychological stress among older adults (20), which may be related to difficulties in emotional regulation and emotional expression (21). In addition, in the context of traditional Chinese culture, some older people may be introverted or accustomed to repressing their emotions to avoid burdening others (22). Therefore, they may not actively express their emotional distress. Effective social support should be provided to this group of older people to encourage them to express their feelings, engage in positive emotional regulation, and avoid excessive emotional repression.

The Extraverted Thinking Group (C2) had a relatively low participation rate of 11.6%, and an average alexithymia score (51.68 ± 5.97). A comparison within the group found that the elderly in this group scored higher on the extraverted thinking dimension than on the other two dimensions. On the surface, the elderly in this group appear to be overly concerned with external matters and may be less attentive to their own feelings. This pattern may be related to the need to cope with the external environment. These elderly people face co-morbidities and the absence of their children, so they need to be independent in arranging daily medication, hospital examinations, etc., which may be associated with greater reliance on extroverted thinking to solve problems (23). In addition, these elderly people often face loneliness and a lack of social support. They may tend to pay attention to the needs and opinions of others and try to use external information to guide their own behaviour and decision-making (24). In the long term, the lack of emotional support may also be a defence mechanism. Older people may distract themselves from emotional distress by paying attention to external things, thereby reducing psychological pressure (25). For this category of elderly people, it may be beneficial to implement structured health goals and tasks to help them establish their identity by utilising their external motivation and logical organisation skills. In addition, effective emotional perception training is also likely to be beneficial (26).

The Emotional Ambiguity Group (C3) had the lowest proportion of participants, at only 10.2%. However, their alexithymia score was the highest among the four categories, at (73.05 ± 4.16) points. This group of older adults had high scores on all three dimensions. Studies have found that cognitive decline and emotional regulation disorders often accompany aging, and co-morbidities and empty-nest status may exacerbate these challenges (27). This group of older adults is in a higher alexithymia state, which may reflect significant difficulties in areas such as emotional expression and recognition. Therefore, personalised interventions to help them effectively recognise and express emotions may be particularly important for improving their mental health.

The proportion of participants in the Good Emotional Adaptation Group (C4) was relatively high, at 39.0%. This group of older adults had the lowest average alexithymia score, at (49.97 ± 7.33) points. This suggests that individuals in this group may have relatively stronger emotional processing abilities compared with the other groups. This may be related to more adaptive cognitive emotion regulation abilities (25). When faced with setbacks, they may demonstrate more effective coping strategies and greater positive psychological resources (28). For this group, dynamic monitoring may be beneficial to help maintain their positive emotional functioning.

This study also showed that age, average monthly household income, the occurrence of acute events in the past year, and the perception of one’s own health status are effective predictors of alexithymia in a potential subgroup of empty-nesters with multiple chronic diseases. Understanding these demographic characteristics can help medical personnel to identify different types of older people early and provide targeted guidance and interventions.

Age was an important factor associated with alexithymia subgroup membership among empty-nesters with multiple chronic conditions. Using the Good Emotional Adaptation Group as a reference, older adults aged 70–79 were more likely to be classified into the Emotional Repression Group, while those aged ≥80 were more likely to be classified into the Emotional Repression Group and the Emotional Ambiguity Group. Advanced age has been reported to be associated with higher levels of alexithymia in older adults (29, 30). As people age, the increased burden of disease, decline in physical functioning, reduced social support, and decreased psychological resilience may be associated with difficulties in emotional recognition and expression, which may in turn be related to higher levels of emotional suppression and alexithymia (11). This finding is consistent with our previous research (25) and further highlights the importance of paying attention to the emotional health and psychological support needs of the very old in clinical and care practice.

Older adults with an average monthly household income of more than 8, 000 CNY were more likely to be classified into the Extraverted Thinking Group, whereas those with an average monthly household income of 3, 000–5, 000 CNY and 5, 000–8, 000 CNY were more likely to be classified into the Good Emotional Adaptation Group. This finding may differ from previous studies (31). One possible explanation is that older adults with a monthly household income of 3, 000–8, 000 CNY may be in a relatively balanced financial situation, which may allow them to meet their basic needs without excessive material and social pressures, and thus may be associated with greater attention to emotional health and psychological adjustment. In contrast, those with a household income exceeding 8, 000 CNY may be more likely to exhibit an externally oriented cognitive style. This may be related to higher expectations and external pressures associated with higher socioeconomic status, particularly in the Chinese socio-cultural context where maintaining social roles and perceived status may place additional demands on older adults, leading individuals to rely more on external factors to evaluate their self-worth and emotional state. This pattern differs from previous findings (32) and may be associated with the specific cultural context, social environment, and income distribution in the present study, such as stronger expectations regarding family responsibility, social role maintenance, and the emphasis on self-reliance among higher-income older adults in the Chinese context.

The occurrence of acute events within the past year was an important factor associated with membership in the Emotional Repression Group. In this study, acute events mainly referred to hospitalisation, acute trauma, and major life changes experienced by older adults within one year. On the one hand, individuals with higher levels of emotional repression may be less likely to detect early problems in a timely manner. On the other hand, emotional distress and stress responses related to acute events may exceed their psychological coping capacity, which may be associated with difficulties in emotional expression and regulation (33). In addition, acute events may disrupt existing psychological coping patterns and may be associated with impaired cognitive emotion regulation abilities, which may further contribute to emotional suppression (34). Therefore, timely identification and appropriate support for emotional distress following acute events may be important for improving emotional well-being and mental health outcomes in older adults.

Perceived health status was associated with alexithymia in both the Emotional Repression Group and the Emotional Ambiguity Group, with older adults in these subgroups showing higher levels of alexithymia. One possible explanation is that uncertainty about health may be associated with increased anxiety and depressive symptoms in older adults (35). Negative perceptions of health and self-aging may be related to emotional distress and may also be associated with cognitive disorganisation (36). When older adults perceive themselves as physically vulnerable, they may experience difficulties in organising and expressing their emotions effectively, which may be associated with emotional blunting. In particular, during the process of health decline, older adults may perceive their physical and psychological states as difficult to understand, which may further exacerbate emotional blunting and confusion (25). Therefore, paying attention to older adults’ perceptions of their health status and providing appropriate emotional support and psychological interventions may be important for improving emotional expression and mental well-being.

## 5 Limitations

There are several limitations to this study. First, the cross-sectional design limits causal inference, and longitudinal studies are needed to explore temporal relationships. Second, the use of convenience sampling and recruitment from six cities within Jiangsu Province may introduce selection bias and limit the generalisability of the findings to other regions.Third, the dichotomization of TAS-20 item responses may have resulted in a loss of information and reduced variability, and the classify-analyze approach may introduce potential classification bias. In addition, data were collected using a self-report measure, which may be subject to recall and social desirability bias. Finally, although a range of demographic variables was included, important psychological covariates (e.g., depression and anxiety) were not assessed, which may limit the comprehensiveness of the analysis.

## 6 Conclusion

Individual differences in alexithymia exist among empty-nesters with multiple chronic conditions. Age, average monthly household income, occurrence of acute events within the past year, and perceived health status were associated with subgroup membership of alexithymia. Given the exploratory nature of the latent class analysis, these findings should be interpreted as identifying potential patterns rather than definitive classifications. Healthcare professionals may consider early screening of alexithymia in this population, with particular attention to the Emotional Repression Group and the Emotional Ambiguity Group. Early identification of high-risk individuals and tailored interventions may be beneficial for promoting active ageing.

## Data Availability

The datasets presented in this article are not readily available because the dataset for this study will not be made publicly available due to ethical restrictions. Specifically, the dataset contains potentially identifiable information from older adults with multiple chronic conditions, and the original informed consent did not include permission for public data sharing. However, de-identified data may be made available from the corresponding author upon reasonable request, subject to approval by the relevant institutional ethics committee. Requests to access the datasets should be directed to BS, Sevenage007@163.com.
