# Supplementary material for: Alexithymia and its subgroup characteristics in Chinese empty-nesters with multiple chronic diseases: an exploration based on latent class analysis
Source: Front Psychiatry. 2026 May 12;17:1779610. doi: 10.3389/fpsyt.2026.1779610 (PMC13202719; doi:10.3389/fpsyt.2026.1779610)
Supplement: Supplementary file 1 [file Table1.docx]

| Y1 | I am often confused about what emotion I am feeling. |
| --- | --- |
| Y2 | It is difficult for me to find the right words for my feelings. |
| Y3 | I have physical sensations that even doctors don't understand. |
| Y4 | I am able to describe my feelings easily. |
| Y5 | I prefer to analyze problems rather than just describe them. |
| Y6 | When I am upset, I don't know if I am sad, frightened, or angry. |
| Y7 | I am often puzzled by sensations in my body. |
| Y8 | I prefer to just let things happen rather than to understand why they turned out that way. |
| Y9 | I have feelings that I can't quite identify. |
| Y10 | Being in touch with emotions is essential. |
| Y11 | I find it hard to describe how I feel about people. |
| Y12 | People tell me to describe my feelings more. |
| Y13 | I don't know what's going on inside me. |
| Y14 | I often don't know why I am angry. |
| Y15 | I prefer talking to people about their daily activities rather than their feelings. |
| Y16 | I prefer to watch "light" entertainment shows rather than psychological dramas. |
| Y17 | It is difficult for me to reveal my innermost feelings, even to close friends. |
| Y18 | I can feel close to someone, even in moments of silence. |
| Y19 | I find examination of my feelings useful in solving personal problems. |
| Y20 | Looking for hidden meanings in movies or plays distracts from my enjoyment. |

Table s1 The full name of the TAS-20 scale
